# Characterization of pathogenic microbiome on removable prostheses with different levels of cleanliness using 2bRAD-M metagenomic sequencing

**DOI:** 10.1080/20002297.2024.2317059

**Published:** 2024-02-22

**Authors:** Tong Wah Lim, Shi Huang, Yuesong Jiang, Yufeng Zhang, Michael Francis Burrow, Colman McGrath

**Affiliations:** a Division of Restorative Dental Sciences, Faculty of Dentistry, The University of Hong Kong, Hong Kong SAR, China; b Division of Applied Oral Sciences and Community Dental Care, Faculty of Dentistry, The University of Hong Kong, Hong Kong SAR, China

**Keywords:** Removable prosthesis, prosthesis cleanliness, metagenomics, pathogenic bacteria, microbial index

## Abstract

**Background:**

The microbiomes on the surface of unclean removable prostheses are complex and yet largely underexplored using metagenomic sequencing technology.

**Objectives:**

To characterize the microbiome of removable prostheses with different levels of cleanliness using Type IIB Restriction-site Associated DNA for Microbiome (2bRAD-M) sequencing and compare the Microbial Index of Pathogenic Bacteria (MIP) between clean and unclean prostheses.

**Materials and Methods:**

Ninety-seven removable prostheses were classified into ‘clean’ and ‘unclean’ groups. All prosthesis plaque samples underwent 2bRAD metagenomic sequencing to characterize the species-resolved microbial composition. MIPs for clean and unclean prostheses were calculated based on the sum of the relative abundance of pathogenic bacteria in a microbiome using a reference database that contains opportunistic pathogenic bacteria and disease-associated information.

**Results:**

Beta diversity analyses based on Jaccard qualitative and Bray-Curtis quantitative distance matrices identified significant differences between the two groups (*p* < 0.05). There was a significant enrichment of many pathogenic bacteria in the unclean prosthesis group. The MIP for unclean prostheses (0.47 ± 0.25) was significantly higher than for clean prostheses (0.37 ± 0.29), *p* = 0.029.

**Conclusions:**

The microbial community of plaque samples from ‘unclean’ prostheses demonstrated compositional differences compared with ‘clean’ prostheses. In addition, the pathogenic microbiome in the ‘unclean’ versus ‘clean’ group differed.

## KEY MESSAGES

- The pathogenic microbiome in the unclean removable prosthesis group tends to be more abundant than that of the clean counterpart among participants with the majority being elders attending a teaching hospital. This finding is worrying because a general decline in systemic health among community-dwelling elders may predispose them to life-threatening diseases.
- By understanding the characteristics of the microbiome of removable prostheses with different levels of cleanliness and the related microbial-infection risks after a comprehensive whole metagenomic sequencing, appropriate prosthesis hygiene care should be emphasized.
- This study introduced a comprehensive and novel method of microbiological investigation of plaque using 2bRAD-M. The taxonomic profile of the microbiome of whole genomes was demonstrated and resolved at the species-level taxonomy for prosthesis biofilms. The biggest advantage of this method included overcoming the DNA sample problems particularly the low-biomass nature of ‘clean’ prosthesis plaque samples. The findings will add to our understanding of the microbiological aspect of removable prosthesis plaque.

## Introduction

Advancements in dentistry and oral self-care globally have resulted in considerable improvements in oral health, with a noted reduction in total tooth loss (edentulism). The number of removable prosthesis wearers continues to increase due to the increasing number and proportion of older people retaining at least ‘some’ teeth. Whilst removable prostheses bring with them enormous benefits for oral and general health, prosthesis-related oral diseases that develop in the absence of proper oral and prosthesis hygiene care are increasing and are of concern. Tooth loss, denture stomatitis, halitosis, periodontal diseases, and dental caries, can have a severe impact on the quality of life of patients [1–6]. While prostheses provide a suitable replacement for missing teeth, they can potentially induce a range of ecological changes within the oral cavity that may contribute to the growth of microbial biofilms, with the majority being bacteria and carrying up to 10^11^ microbes per milligram [7,8]. However, most microbiological studies about removable prostheses have historically focused on fungi, particularly *Candida albicans* resulting in a fundamental gap in the prosthesis microbiome composition [9,10].

To date, microbiological studies of the removable prosthesis microbiome composition have been limited by using culture-based assessments and focus mainly on fungi composition [11]. Studies on the microbiome of removable prostheses adopted 16S rRNA-based sequencing and have only been identified up to the genus-level taxonomy [7]. The microbiome on removable prostheses is complex with higher *Candida spp* colonization compared to the dental biofilm. However, the dental biofilm was found more diverse than biofilm on removable prostheses [8,11]. O’Donnell et al. found that bacterial microbiomes between removable prostheses, palatal mucosa, and dental plaque formed distinct clusters and were compositionally different and the removable partial prosthesis biofilm samples were found to exhibit a higher microbiome diversity than the complete prostheses [8]. The predominant microbiota on removable prosthesis materials included polymethylmethacrylate resin and cobalt-chromium alloys characterized by *Streptococci spp*, *Staphylococci spp*, and fungi, while *Lactobacillus*, *Actinomyces*, *Atopobium*, and *Scardovia* are more prevalent in prosthesis biofilm than mucosal samples [8,12,13]. Moreover, when the prostheses are taken out of the mouth, they can easily be contaminated by unhygienic environments, leading to the introduction of pathogenic microorganisms into the oral cavity that are not normally found there. The materials used to fabricate removable prostheses have the properties of being abiotic, hard, and non-shedding but rough on intaglio surfaces, making the prostheses more prone to microbial adhesion and forming a distinct plaque microbiome with less exposure to the host immune response [8,11]. This susceptibility to microbial colonization is further exacerbated by the surface energy, hydrophobicity, chemistry, and complex topography of the prostheses [11–15].

Recently, potentially harmful microorganisms from prosthesis biofilm have been suggested to be associated with significant health problems, such as bacterial endocarditis, gastrointestinal infections, and respiratory diseases [11,16–22]. Of prime concern is that removable prostheses can harbor and facilitate the growth of a diverse array of gram-positive and gram-negative bacterial microorganisms, as well as fungi, leading to systemic infections, particularly among frail older adults with comorbidities [23]. *Moraxella lacunata* and *Neisseria perflava*, both of which are gram-negative cocci pathogenic bacteria, have been identified as capable of proliferating and thriving on prosthesis surfaces. It is important to note that *Neisseria perflava* may lead to severe medical conditions such as endocarditis, septicemia, and meningitis [24,25]. *Acinetobacter spp*. and *Burkholderia cepacia* are known to cause nosocomial infections, which are infections acquired in healthcare settings. Venkataraman et al. [26] identified that the microbes residing intraorally are the primary driver of the lung microbiome. Therefore, the potential for respiratory bacteria including *Staphylococcus aureus, Streptococcus pneumoniae, Pseudomonas aeruginosa, Haemophilus influenzae B, Streptococcus pyogenes*, and *Moraxella catarrhalis*, residing on prosthesis surfaces could be the source of microorganisms aspirated into the lung [15]. A variety of gram-positive bacteria have been identified on removable prosthesis surfaces, particularly *Staphylococcus aureus, Staphylococcus epidermidis, Arcanobacterium heamolyticum*, and *Actinomyces spp*., and these have been reported to be associated with various human health conditions (7,8). Among the different yeasts and fungi that were identified, including *Aspergillus spp., Candida albicans, Candida glabrata, Candida dubliniensis, Candida parapsilosis, Candida paratropicalis, Candida krusei*, and *Trichosporon mucoides*, some have been shown to be the principal species responsible for inflammatory pathology [27–30].

Whole metagenomic and high-throughput sequencing approaches have allowed comprehensive compositional analysis of the microbial community, including 16s rRNA-based sequencing and metagenomic shotgun sequencing. More recently, Type IIB Restriction-site Associated DNA sequencing for Microbiome (2bRAD-M) was introduced to profile bacterial and fungal communities in a cultivation-independent way, providing quantitative and qualitative data by demonstrating and resolving species-level taxonomy [31]. This sequencing method may discover new microbial species in the prosthesis biofilm. Additionally, this method allows for overcoming DNA sample problems, including low biomass, heavily degraded microbiomes, or contamination of the host DNA [31]. This method also helps to reduce the bias of the sequencing technique and expand the boundary of microbiome profiling of low-biomass plaque samples. This potentially offers a cost-effective and high-resolution sequencing technique that will be beneficial for analyzing low-biomass plaque samples from clean prostheses. In addition, screening pathogenic bacteria using the Microbial Index of Pathogenic bacteria (MIP), a type of microbiome-based index within the plaque biofilm using this high-throughput sequencing method allows the risk assessment of oral and systemic health diseases, potentially controls the transmission of pathogens, and reduces the risk of diseases [32–34]. The microbiome-based indices were developed for the skin health [35], gut microbiome [36], dental caries [37], and periodontal diseases [38]. The MIP can be calculated as the sum of the relative abundance of opportunistic pathogenic bacteria in a microbial community according to 300 published categories of opportunistic pathogenic bacteria by the Chinese Center for Disease Control and Prevention [32,39].

Currently, there are very few published removable prosthesis microbiome studies, particularly those involving sample collection directly from the prosthesis itself. It is worth noting that the majority of removable prosthesis wearers are older adults, who may struggle to maintain good oral hygiene care because of decreased manual dexterity and cognitive issues [40]. Additionally, their fragile oral tissues are more prone to colonization by opportunistic microbiota, consequently, their susceptibility to oral and systemic diseases increases [12]. At present, the complete microbiological profile up to the species-level taxonomy and the MIP of both clean and unclean removable prostheses are unknown. Therefore, this study aimed to characterize the microbiome of removable prostheses with different levels of cleanliness using a high-resolution metagenomic sequencing method (2bRAD-M) and compare the MIP between clean and unclean prostheses.

## Materials and methods

### Sample size determination and participant recruitment

The sample size was confirmed with the rarefaction curve (Shannon diversity versus number of samples sequenced) showing that adequate samples have been collected for covering oral species-level diversity at the population level. Following ethics approval (IRB Reference Number: UW22–256), Institutional Review Board of the University of Hong Kong/Hospital Authority Hong Kong West Cluster, a total of 97 removable prosthesis participants who met the inclusion criteria were recruited from patients attending a teaching dental hospital using a convenience sampling method. The inclusion and exclusion criteria were being aged 18 or older, being able to comply with the study protocol and provide informed consent, and wearing removable prostheses for at least 3 months. Participants who had received antimicrobial or antifungal treatment in the past month, taken antibiotics in the past 4 weeks, taken steroids in the past 6 months, undergone radiotherapy or chemotherapy, and rinsed their mouth with mouthwashes prior to sampling were excluded from the study. Prosthesis image acquisition and plaque sample collection were conducted at the Prosthodontic Clinic and transferred immediately to the Central Research Laboratories in the same building. All items used in this assessment were autoclaved prior to sample collection to ensure a clean and controlled environment during the sample collection process and reduce the risk of introducing contaminants. Following on the clinical variables were examined by a trained and calibrated prosthodontist (T.W.L.).

### Prosthesis image acquisition and percentage plaque area coverage quantification

Prosthesis plaque area quantification was determined using a semi-automated planimetric assessment [41]. Standardized biofilm staining protocols were performed. Removable prostheses were removed from the participants’ mouths and rinsed under running water held by a sterilized tweezer. The fitting surface of the prosthesis biofilm was gently stained with a sterilized disclosing agent (Ci double plaque checker®, C.I. Medical Co., Ltd. Ishikawa, Japan) using a sterilized swab. The prosthesis was then positioned on a piece of sterilized white A4 paper in a photo lightbox. All color images were taken using standardized protocols and camera settings (ISO: 100; exposure: 200; aperture: 22; white balance: flash). Ninety-seven images were uploaded for processing and analysis. The hue, saturation, and value parameters were adjusted by thresholding to segment the stained biofilm pixels. The percentage plaque area coverage (PPC) was calculated automatically. The whole biofilm area coverage quantification protocol was performed by one examiner (prosthodontist). The PPC was obtained and grouped as either ‘clean’ (PPC <25%) or ‘unclean’ (PPC ≥25%) prostheses [40,41].

### Prosthesis plaque sample collection and processing

The recommended materials and methods of O’Donnell et al. [8] was followed for biofilm sample collection for metagenomic analyses. Prostheses were placed in sterilized bags containing 50 mL sterilized phosphate-buffered saline and placed in an ultrasonic bath for 15 mins at 45 kHz to remove the adherence biofilm. The prosthesis sonicate was transferred and centrifuged at 9880 rpm (14,000 g) for 10 mins. The remaining plaque pellet was resuspended in 180 μL of 20 mM Tris-Hcl; 2 mM EDTA; 1.2% Triton with 20 μL of 20 mg/ml lysozyme and incubated overnight at 37°C. 20 μL proteinase K extraction buffer was added and mixed by vortexing. The samples were incubated at 56°C for 2 hours followed by 95°C for 15 mins. Ethanol (200 μl) was added to the sample and mixed by pulse-vortexing for 15 seconds. Then, the DNA was extracted using QIAmp Mini DNA Extraction Kit (Qiagen GmbH, Germany). DNA concentration was quantified by the Qubit 2.0 Fluorometer (Life Technologies, Carlsbad, California, US), and the samples were then stored at −70°C.

### Type IIB restriction-site associated DNA sequencing (library construction and metagenomic sequencing)

All samples were sequenced using the 2bRAD method [31] at Qingdao OE Biotech Co., Ltd. (Qingdao, China). The extracted DNA was digested using 4 U Type IIB restriction enzyme (BcgI) for three hours at 37°C. The reaction mixture using 10 μL digested product, T4 DNA Ligase Buffer, 1 mM ATP (NEB), 0.2 μM of adaptors (Ada1 and Ada2), and 800 U of T4 DNA ligase (NEB) was prepared for 16 hours of the ligation reaction. Following on the enzyme heat inactivation for 20 minutes at 65°C. The amplification for DNA sequencing through Polymerase Chain Reaction (PCR) in a reaction volume of 40 μL. The PCR mixture contained 7 μL of ligated DNA, 1 × Phusion HF buffer, 0.1 μM of primers for Illumina, 0.3 mM dNTP, and 0.4 U of Phusion high-fidelity DNA polymerase (NEB). The PCR reactions were performed in a DNA Engine Tetrad 2 thermal cycler (Bio-Rad) for 16 to 28 cycles under the following conditions: initial denaturation at 98°C for 5 seconds, followed by annealing at 60°C for 20 seconds, extension at 72°C for 10 seconds, and a final extension at 72°C for 10 minutes. The target bands of approximately 100 bp were excised from the 8% (wt/vol) polyacrylamide gel, and the DNA was diffused in nuclease-free water for 12 hours at 4°C. Barcodes unique to each sample were incorporated through PCR using primers containing platform-specific barcodes. The 40 µl PCR mixture consisted of 50 ng of PCR product extracted from the gel, 0.2 µM of each primer, 0.6 mM dNTP, 1× Phusion HF buffer, and 0.8 U of Phusion high-fidelity DNA polymerase (NEB). The products were purified using the QIAquick PCR purification kit (Qiagen, Valencia, CA). Following on, the purified products were sequenced using the Illumina Novaseq 6000 platform (Adaptors and primers sequences are listed in Appendix 1).

Raw sequences were processed and selected using the FastQ Quality Control tool. Raw reads underwent a filtration procedure to extract the specific digested fragments (enzyme reads) which were recognized by the BcgI restriction enzyme. Clean reads were obtained after i) discarding reads with greater than 8% unknown bases and ii) filtering out low-quality reads containing more than 20% of low-quality bases with a quality score below Q30. With 2bRAD-M sequencing data (32-bp long reads), the species-resolved compositional profile for each prosthesis biofilm sample was obtained from the bioinformatic pipeline (built-in Perl scripts: https://github.com/shihuang047/2bRAD-M) [31]. This bioinformatic pipeline relies on a specialized 2bRAD tag database that includes taxa-specific tags obtained from a comprehensive collection of 173,165 microbial genomes from the NCBI RefSeq database, encompassing bacteria, archaea, and fungi. For each microbiota, microbial species were identified based on a prebuilt 2bRAD species-specific marker database. For each species, the abundance was estimated based on the sequencing coverage of its species-specific markers [31].

### Statistical analysis

The relative abundance in 2bRAD was calculated using the below formula. A G score threshold of 5 was determined to control false positives. The average read coverage of 2bRAD markers for each species was first determined (representing the number of individuals belonging to a species in a sample). The ratio (relative abundance) was confirmed by dividing this value with the total number of individuals from all detected species in a sample.

$$\mathrm{Relative}\;\mathrm{abundanc}e_{\mathrm{species}\;i}=\frac{S_{i}/T_{i}}{\sum_{i=1}^{n}S_{i}/T_{i}}$$

*S*: The number of reads assigned to all 2bRAD markers of *species i* in a sample

*T*: The number of all theoretical 2bRAD markers of *species i*

The taxonomic abundance profiles were used to calculate Alpha diversity indices, including the Simpson index, Shannon index, and Chao 1 index. These calculations were carried out using the R programming language, specifically leveraging the functionalities provided by the ‘vegan’ package. For Beta diversity estimation, the Jaccard distance, Bray-Curtis distance, and Euclidean distance matrices were calculated [42]. These calculations were also performed using the ‘vegan’ package in R software (version 4.2.1), and the results were visualized using Principal Coordinate Analysis (PCoA). Statistical analyses were carried out using R software (version 4.2.1). The variations in the Alpha and Beta diversity between the two groups were determined using the Wilcoxon signed-rank test and permutational multivariate analysis of variance (Permanova test), respectively [43]. In addition, the ratios between the relative abundance of human and bacterial DNA for both groups were compared using the Wilcoxon rank sum test after ultra-low human abundances (values <1E–5 or its corresponding bacterial abundance equals 1) to zeros. The data were logarithm-transformed with a base of 10 (log_10_) to facilitate the observation of intergroup differences (zeros will be transformed to 1E–10 for logarithm transformation). The significance level was set at 0.05.

Species-level abundance profiles were obtained to determine the MIP for each prosthesis microbiota using the below formula. The MIP was calculated as the sum of the relative abundance of all opportunistic pathogenic bacteria in a microbial community according to 300 published categories of opportunistic pathogenic bacteria by the Chinese Center for Disease Control and Prevention, ranging from 0 to 1 [32,34]. The MIP was calculated using the software ‘Microbial Index of Pathogenic Bacteria (MIP)’ (https://github.com/qdu-bioinfo/mip) [32]. The variation in MIP was determined using an independent t-test for these two groups after the data had undergone square root transformation. Following on, the size and design of the prosthesis (cross-arch) were taken into consideration for the MIP analysis. The significance level was determined at 0.05 and analyzed using IBM SPSS Statistics 27.0.

$$\mathrm{MIP}=\frac{\sum_{i=1}^{M}\mathrm{pathogen}s_{i}}{\sum_{j=1}^{N}m_{j}}$$

*M*: The number of pathogens in a sample

*N*: The number of all microbes identified in a sample

*m*: The relative abundance of a microbe in a sample

## Results

### Participants characteristics *[40]*

A total of 97 removable prosthesis plaque samples (97 participants) from 41 males and 56 females were collected in this study (Table 1). The mean ± standard deviation (SD) age of the participants was 68.97 ± 11.98 years. Of these prostheses, 53 (54.64%) were maxillary and 44 (45.36%) were mandibular. Most of the prostheses were of partial design type (83.51%) and 16 (16.49%) were complete prostheses. Age, sex, dental arch with prosthesis, and classification of prosthesis were not significantly associated with prosthesis cleanliness (all, *p* > 0.05). Slightly less than half of the prostheses (47.42%) were polymethylmethacrylate resin and 51 (52.58%) were metallic framework (cobalt-chromium). The effect of prosthesis materials, classification of the prosthesis, and presence of denture stomatitis had no significant influence on microbial diversity as shown by Appendix 2. The mean ± SD PPC of all the prostheses was 24.79 ± 19.78%. There were slightly more ‘clean’ than ‘unclean’ prostheses (56.7% vs 43.3%) as reported by Lim et al. [40].

**Table 1.** Sociodemographic and prosthesis-related information.

Factor	Clean prosthesis (PPC < 25%)	Unclean prosthesis (PPC ≥ 25%)	Total
Age			
<65-year-old	18 (56.25)	14 (43.75)	32 (32.99)
≥65-year-old	37 (56.92)	28 (43.08)	65 (67.01)
Sex			
Man	24 (58.54)	17 (41.46)	41 (42.27)
Woman	31 (55.36)	25 (44.64)	56 (57.73)
Endocrine disease			
Yes	13 (50.00)	13 (50.00)	26 (26.80)
No	42 (59.15)	29 (40.85)	71 (73.20)
Cardiovascular disease			
Yes	30 (60.00)	20 (40.00)	50 (51.55)
No	25 (53.19)	22 (46.81)	47 (48.45)
Gastrointestinal disease			
Yes	6 (50.00)	6 (50.00)	12 (12.37)
No	49 (57.65)	36 (42.35)	85 (87.63)
Dental arch with prosthesis			
Maxilla	30 (56.60)	23 (43.40)	53 (54.64)
Mandible	25 (56.82)	19 (43.18)	44 (45.36)
Prosthesis material			
Polymethylmethacrylate resin	33 (71.74)	13 (28.26)	46 (47.42)
Metallic framework (cobalt-chromium)	22 (43.14)	29 (56.86)	51 (52.58)
Classification of prosthesis			
Removable partial prosthesis	46 (60.71)	35 (39.29)	81 (83.51)
Removable complete prosthesis	9 (56.25)	7 (43.75)	16 (16.49)
Denture stomatitis			
Yes	8 (34.78)	15 (65.22)	23 (23.71)
No	47 (63.51)	27 (36.49)	74 (76.29)

Values are presented as numbers and percentages, n (%).

PPC, Percentage plaque area coverage.

### Diversity of the clean and unclean removable prosthesis microbiome

**Figure 1.**
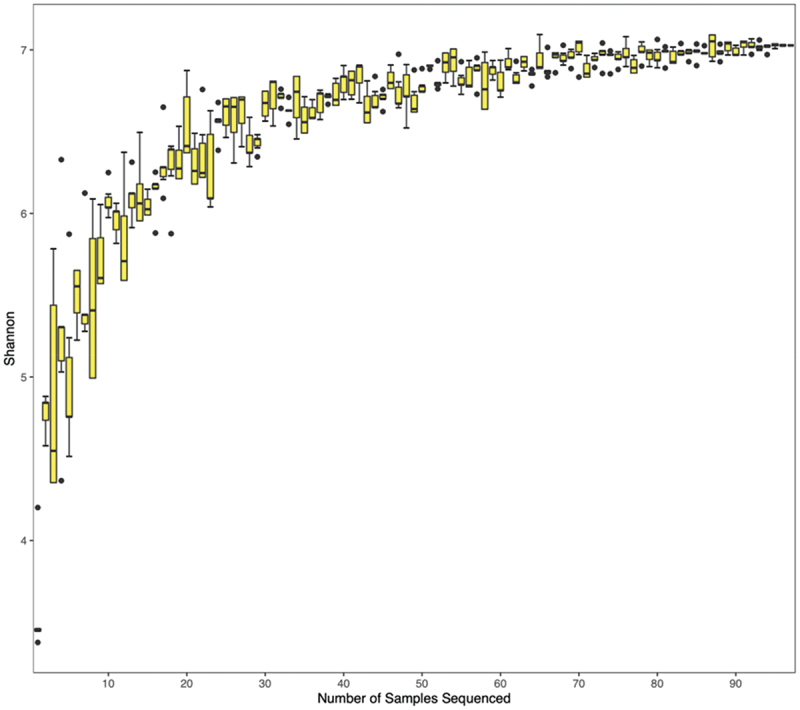
Shannon diversity with the number of samples sequenced revealed adequate sample size for species detection.

Ninety-seven samples were sequenced and 7,996,330 raw reads underwent the filtering process. A total of 3,560,707 enzyme reads were extracted and 3,262,705 clean reads were obtained with an average of 33,636 reads per sample. Ultimately, this study detected 24 phyla, 38 classes, 83 orders, 161 families, 547 genera, and 1989 species. The Shannon diversity with the number of samples sequenced showed the sample size was adequate for this study because the curve was flattened towards the end, which indicated the diversity of species in the community did not increase with an increase in the sample size (Figure 1).

**Figure 2.**
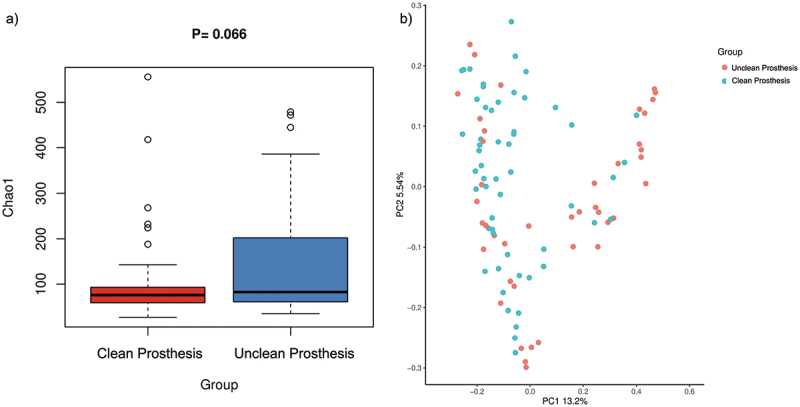
a) alpha diversity analysis (Chao 1) revealed no significant difference in microbial richness between clean and unclean prostheses (*p* = 0.066). b) beta diversity analysis, Principal Coordinate analysis based on the Jaccard (PERMANOVA; *R*^*2*^ = 0.026, *p* = 0.001) distance matrix.

The overall microbial evenness and richness in both groups (clean and unclean prostheses) showed no statistically significant difference (Figure 2a) according to Chao 1 (*p* = 0.066), Shannon (*p* = 0.396), and Simpson (*p* = 0.933) indices. However, Beta diversity analyses based on Jaccard qualitative and Bray Curtis quantitative distance matrices revealed significant differences in the microbial community structures between the two groups. The differences in microbial community between clean and unclean prostheses were further confirmed by the Permanova test (Jaccard: *R*^*2*^ = 0.026, *p* < 0.001; Bray Curtis: *R*^*2*^ = 0.015, *p* = 0.037). In the PCoA (Figure 2b), the samples from clean prostheses were more located on the left side of the plot. However, they presented with some overlapping with the unclean prosthesis group on the right side.

### Relative bacterial density in clean and unclean prostheses

**Figure 3.**
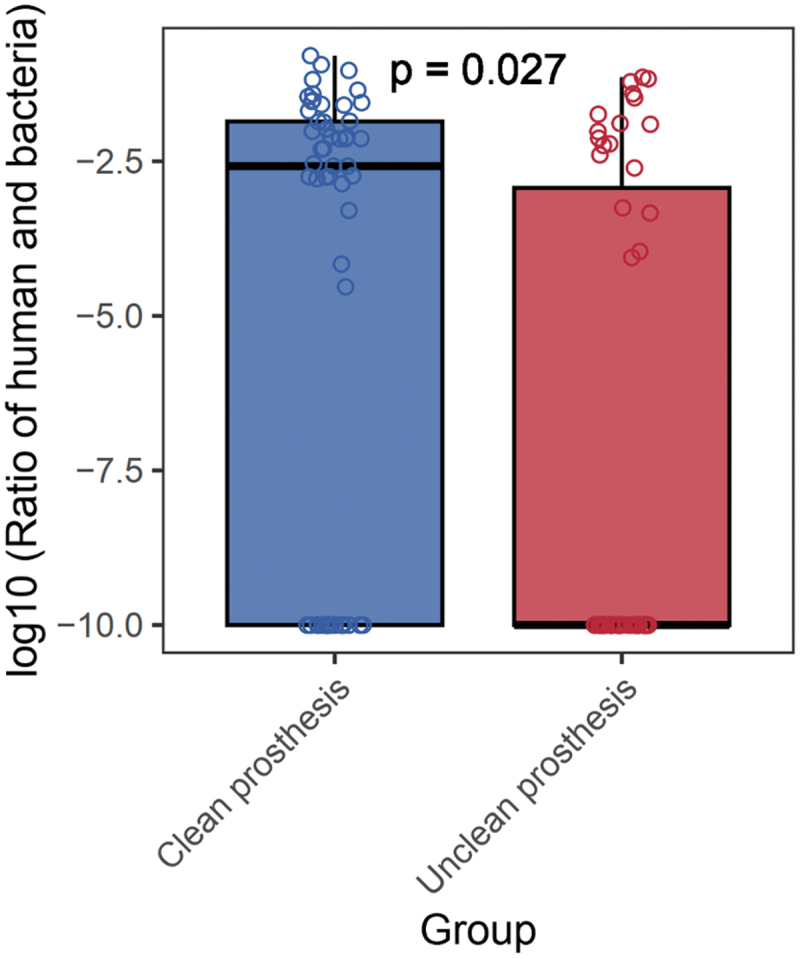
Boxplot of the ratios between the relative abundance of human and bacterial DNA.

In this study, a low relative abundance of fungi (*C. albicans*, *C. glabrata*, *C. dubliniensis*, *C. tropicalis*) and no archaeal signals were detected in any of the samples. In addition, the proportion of relative abundance of fungi in comparison to bacteria was low. The fungi-bacteria ratio had no statistically significant difference between clean and unclean prostheses(*p* = 0.179, Mann-Whitney test). The 2bRAD sequencing retains human DNA information. Therefore, the ratios of human DNA to bacterial DNA in the two groups were computed. The unclean prosthesis group demonstrated a significantly lower proportion of human DNA content (*p* = 0.027) and it can be postulated that this represents a higher abundance and more complex microbial community than the clean prostheses (Figure 3 and Appendix 3).

### Microbial composition colonizing on removable prosthesis surfaces

**Figure 4.**
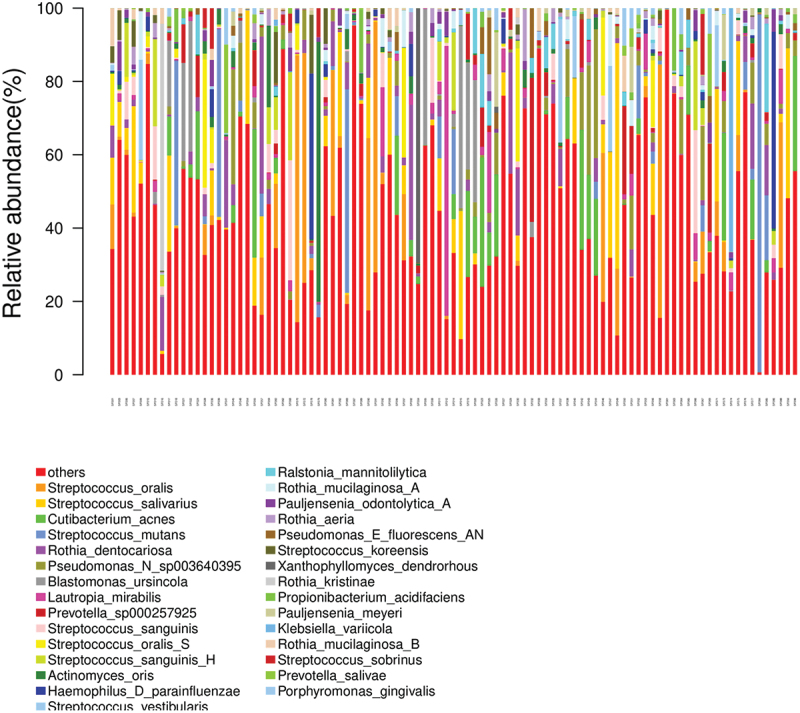
Top 30 most abundant species in the removable prosthesis biofilm.

The taxonomic distributions of this study showed that the top five most abundant phyla included Firmicutes, Proteobacteria, Actinobacteria, Bacteroidetes, and Firmicutes_C. Bacilli, Actinomycetia, and Gammaproteobacteria were the top three classes found on the prosthesis surfaces. The five most predominant genera among 547 genera were Streptococcus, Rothia, Prevotella, Cutibacterium, and Neisseria. At the species level, *Streptococcus oralis*, *Streptococcus salivarius*, *Cutibacterium acnes*, *Streptococcus mutans*, and *Rothia dentocariosa* were the five most abundant bacteria. Nevertheless, among the top 30 species colonizing prosthesis surfaces, *Streptococcus oralis, Streptococcus mutans*, *Streptococcus sobrinus*, *Ralstonia mannitolilytica*, *Haemophilus parainfluenzae*, *Klebsiella variicola*, and *Porphyromonas gingivalis* were recognized pathogens (Figure 4).

**Figure 5.**
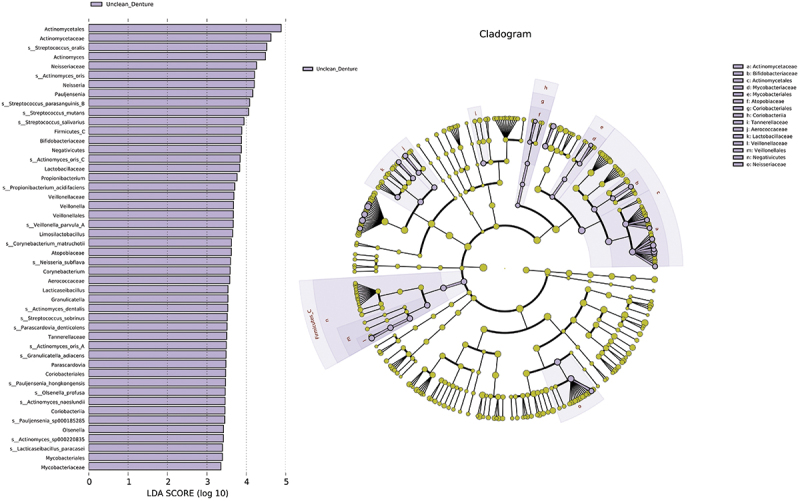
The LEfSe analysis indicated a higher abundance of 48 taxa in the unclean prosthesis group than in clean prostheses. The brightness of each point was proportional to the size of its effect.

The microbial communities at the species level were compared between clean and unclean prostheses using LEfSe analysis (Figure 5). There were 48 taxa that exhibited relatively higher abundances in the unclean prosthesis group [LDA score (log 10) > 3]. In this group, there was a significant enrichment of cariogenic bacteria including *Streptococcus mutans, Streptococcus sobrinus*, and *Streptococcus oralis*. Additionally, *Streptococcus oralis* and *Streptococcus salivarius* were identified as the top two species important for random forest model accuracy for prosthesis cleanliness prediction, with a mean decrease in accuracy of more than 4.0 (Appendix 4). The top 10 bacterial species with the largest relative abundance differences between clean and unclean prosthesis groups are shown in Appendix 5. The indicator analysis showed the ability of *Ralstonia mannitolilytica* and *Actinomyces oris* to be an indicator of clean and unclean prosthesis groups, respectively (Appendix 6).

### Microbial index of pathogenic bacteria

The mean and SD of the MIP for all 97 samples was 0.41 ± 0.28, with MIP ranging from 0 to 0.98. For comparison between the two groups, the mean and SD of the MIP for unclean prostheses (0.46 ± 0.25) was higher than for clean prostheses (0.37 ± 0.29). However, the difference was not statistically significant (*p* = 0.052). After taking the size and design of the prosthesis into consideration (unilateral sectional removable prosthesis was excluded), the mean MIP for unclean prostheses increased by 0.01 and resulted in a statistically significant difference compared with clean prostheses (*p* = 0.029).

## Discussion

Removable prostheses are colonized by relatively stable yet complex and dense polymicrobial consortia of bacteria and yeasts. The presence of pathogenic microorganisms in the prosthesis biofilm is of great interest to public health and safety, particularly to the prosthesis wearers who are mainly older adults who are often medically compromised [7,11]. Prosthesis wearers are exposed to the microbial community within the oral cavity through tissue contact, swallowing, and respiration, increasing the risk of oral and systemic health diseases [15,16]. Therefore, the comprehensive microbiome profile of removable prostheses using high-resolution whole metagenomic 2bRAD sequencing is crucial and urgently needed to identify the species diversity and investigate the complexity of the removable prosthesis biofilm with different levels of cleanliness. In addition, the MIP is particularly useful for assessing and predicting prosthesis cleanliness for health status trends [32,33]. The present study reported that bacterial microbiomes between clean and unclean removable prostheses are compositionally different and the unclean prosthesis group had significantly higher MIP compared to the clean prosthesis group after considering the prosthesis design. Therefore, the research hypotheses were accepted.

Removable prostheses provide new ecological niches and potentially induce alterations in the structure and composition of microbial communities. The microbial evenness and richness in both groups (clean and unclean prostheses) showed no statistically significant differences by all indices, potentially obscured by the resilience, dynamics of the oral microbiome, or diverse individual variables. However, a clear trend of increased Chao index (species richness) in the prosthesis microbiome was demonstrated on the unclean prosthesis surfaces. In addition, the Beta-diversity analysis based on the Jaccard distance and Bray Curtis matrices showed a significant difference in separation between clean and unclean prosthesis groups, indicating a distinction between the two microbial communities. The minor overlapping without well-separated clusters as shown in the PCoA plot can be still considered reasonable as the bacterial profiles on the removable prostheses may maintain some similarity. Nevertheless, the clean and unclean prostheses had their unique bacterial profiles which were different from each other including the presence of individual species and their relative abundance. These data likely indicate a complex relationship between removable prosthesis microbial communities and prosthesis cleanliness. The presence of dysbiosis in the microbial community and disruption of the host-microbial homeostasis by some pathogenic bacteria may result in oral and systemic diseases [44,45]. Therefore, prosthesis-related diseases that develop in the absence of proper oral and prosthesis hygiene care are of concern.

Previously published microbiological studies of the removable prosthesis microbiome composition have been limited by using culture-based assessment and focused mainly on fungi, particularly the association between *Candida spp* with denture stomatitis [10,28,46]. The introduction of molecular techniques allows a comprehensive study of the removable prosthesis microbiome, particularly microorganisms that are not culturable. The oral microbiome carries up to 700 species of bacteria [47,48]. In contrast, this study detected 1989 microbial species (bacteria and fungi). This finding may be explained by the Genome Taxonomy Database (GTDB R202) was employed in this study to analyze the metagenomic data. GTDB R202 is a comprehensive microbial genome to date including 258,406 genomes organized into 47,894 species clusters (https://gtdb.ecogenomic.org/stats/r202) [49]. Notably, GTDB R202 contains a high number of curated genomes and is regularly updated. GTDB has also established a new and universal taxonomy system based on genome-wide distance, collected a comprehensive set of microbial genomes from NCBI RefSeq, and renamed the microbes according to their between-genome similarity. Whereas the Human Oral Microbiome Database (HOMD) [48] primarily focuses on collecting 16S rRNA genes in the oral microbiota using the conventional NCBI taxonomy. In addition, as whole-genome sequencing methods have progressed, studies have been able to delve deeper into the microbial diversity present in the human oral microbiota. This has led to a better understanding of why the content of the HOMD has expanded (*N* = 774 species). A recent study identified a total of 3589 oral species-level genome bins assembled from 4154 (3346 new samples) meta-analyzed oral metagenomes [50], most of which have never been characterized in the previous studies. This finding is also consistent with that of He et al. [51]. They adopted the same 2b-RAD sequencing technique for oral samples and detected more than 1900 microbial species in the saliva. At present, very limited published removable prosthesis microbiome studies exist that have used high-throughput techniques and all of them adopted the 16S-based sequencing [8,52,53]. Nevertheless, this technique identified microbial communities and structures present in the prostheses and has only been identified up to the genus-level taxonomy [8]. Among them, only five studies collected plaque samples solely from the removable prostheses whereas the remaining six combined the samples from the mucosa, tongue, and saliva [7]. Therefore, the main strength of this study is its investigation of the general characteristics of microbiome alterations caused by prosthesis cleanliness, using a high-throughput 2bRAD method and species-level taxonomy resolution on a large sample size.

Bacilli and Actinomycetia were the two predominant classes identified on prosthesis surfaces in this study. At the genus level, Streptococcus, Rothia, Prevotella, Cutibacterium, and Neisseria were dominant in the present study. These findings are consistent with O’Donnell et al. [8] and Yitzhaki et al. [54], who reported Streptococcus and Rothia were the most abundant genera. Among the 97 samples tested, *Streptococcus mutans*, *Streptococcus sobrinus*, *Ralstonia mannitolilytica*, *Haemophilus parainfluenzae*, *Klebsiella variicola*, and *Porphyromonas gingivalis* were the top found pathogenic bacteria. *Streptococcus oralis, Streptococcus mutans, Streptococcus sobrinus*, and *Porphyromonas gingivalis* are the main cariogenic and periodontopathic pathogens, involved in the pathogenesis of dental caries and periodontal diseases [5,6]. For systemic health, *Ralstonia mannitolilytica, Haemophilus parainfluenzae*, *Klebsiella variicola* are opportunistic pathogens causing bloodstream infections, respiratory tract infections, urinary tract infections, and meningitis [19–21]. As expected, unclean prostheses had a higher abundance of 48 taxa than clean prostheses through LEfSe analysis. The result showed significant enrichment of cariogenic bacteria, suggesting a high prevalence of caries incidence in patients wearing removable prostheses with poor prosthesis hygiene [3].

The present study found that the mean MIP for unclean prostheses was significantly higher than for clean prostheses, suggesting the risk of oral and systemic health diseases. The susceptible human organs associated with the pathogenic bacteria in the MIP include oral and sensory organs, skin, cardiovascular, urogenital, gastroesophageal, central nervous, and other systems [32]. Furthermore, the unclean prosthesis group demonstrated a significantly higher proportion of bacterial DNA content in relation to human DNA, suggesting bacterial density of unclean prostheses was higher, possibly increasing microbial complexity and the susceptibility to oral and systemic diseases. This finding is consistent with a study reporting the high ratio of bacteria to human cells in the periodontitis group compared to the healthy controls [55]. However, the authors have no intention to make predictive modeling of any oral and systemic diseases in this study. Thus, longitudinal studies with longer follow-up periods evaluating the human health between clean and unclean prostheses using the MIP are recommended to overcome the limitations of the existing evidence. In contrast, Banerjee et al. [56] emphasized the role of keystone taxa, which can be defined as low abundant but ecologically significant taxa. A recent review reported the emerging evidence on the association between oral microbial dysbiosis and tumorigenesis [57], emphasizing that microbial activity and virulence of pathogens may contribute a significant ecological role in carcinogenesis and the low relative abundance may not adequately reflect the impact of keystone pathogens on the health status of patients. Thus, the importance of functional differences between pathogens irrespective of their abundance for understanding their ecological roles and impact on health should not be underestimated [56]. Nevertheless, it is important to recognize the significance of the observed high abundance of potential pathogens within a human body habitat, as it raises valid concerns regarding the overall health of the host.

The present study recruited mainly community-dwelling elders with diverse individual variables including wearing different designs of removable prostheses, which represents the main limitation of this study. The justification for selecting such a diverse group of participants was that the plaque samples of this study were solely collected from the adherent plaque biofilm on the removable prostheses and the saliva microbiome on the prosthesis surfaces was also rinsed away during the sample collection [58]. In addition, the plaque-disclosing agent’s impact on the sequencing results remains uncertain but this protocol is supported by the methodologies employed in previous studies [59,60]. Notably, within its limitations, the prosthesis materials, denture designs, and denture stomatitis were taken into consideration during the microbiome analysis. However, the microbial diversity of plaque samples demonstrated no difference between groups (Appendix 2). This is in line with Mukai et al. [61]. In addition, the present study sequenced a relatively large sample size to compensate for individual and environmental variations and attempted to explore the general profile of microbiome alterations affected by prosthesis cleanliness.

## Conclusions

Among a community-dwelling sample of participants attending a teaching hospital, approximately half had unclean prostheses. The microbial community of plaque samples from unclean prostheses demonstrated a clear distinction compared with clean prostheses. In addition, a trend was also observed for the increased MIP in the unclean group in comparison with the clean counterpart. The key findings of this study have implantation to dental and medical fields and with potential impact on aged care.

## Supplementary Material

Appendix 3.pdf

Appendix 1.pdf

Appendix 6.pdf

Appendix 4.pdf

Appendix 2.pdf

Appendix 5.pdf
