# Supplementary material for: Characterization of pathogenic microbiome on removable prostheses with different levels of cleanliness using 2bRAD-M metagenomic sequencing
Source: J Oral Microbiol. 2024 Feb 22;16(1):2317059. doi: 10.1080/20002297.2024.2317059 (PMC10896157; doi:10.1080/20002297.2024.2317059)
Supplement: Appendix 3.pdf [file ZJOM_A_2317059_SM6762.pdf]

Table 1. Bacteria-human ratio for all 97 samples.

Reference number	Bacteria	Human	Ratio
SF001	1	4.48E-14	4.48E-14
SF002	0.991944344	0.00772133	0.007784035
SF003	1	1.57E-114	1.57E-114
SF004	0.304915364	0.001546091	0.005070557
SF005	0.994178406	0.001360903	0.001368872
SF006	0.999950586	1.63E-28	1.63E-28
SF007	1	6.05E-50	6.05E-50
SF008	0.999465821	5.36E-21	5.36E-21
SF009	0.997098901	0.002459458	0.002466614
SF010	1	5.23E-41	5.23E-41
SF011	0.979628199	0.020268843	0.020690343
SF012	1	3.15E-19	3.15E-19
SF013	0.999993643	3.97E-06	3.97E-06
SF014	0.972643109	0.004839166	0.004975274
SF015	0.994573681	0.002870131	0.00288579
SF016	0.999999705	3.89E-20	3.89E-20
SF017	0.984710236	0.00942147	0.009567759
SF018	1	5.02E-38	5.02E-38
SF019	0.998165701	0.001778786	0.001782054
SF020	0.974449703	0.025535505	0.026205052
SF021	0.987205331	0.012787839	0.012953576
SF022	0.994331462	0.005664333	0.005696625
SF023	0.897923917	0.040138027	0.044700922
SF024	0.962315312	0.037612232	0.039085143
SF025	0.854967821	0.137754067	0.161121932
SF026	0.950627568	0.03334453	0.035076334
SF027	0.999976194	4.01E-30	4.01E-30
SF028	1	1.94E-36	1.94E-36
SF029	0.998327117	0.00164384	0.001646595
SF030	1	5.74E-23	5.74E-23
SF031	0.999998594	6.96E-10	6.96E-10
SF032	0.914821438	0.085178562	0.093109495
SF033	0.99978823	1.07E-09	1.07E-09
SF034	0.964987795	0.02951254	0.030583329
SF035	0.989093329	6.81E-05	6.89E-05
SF036	1	8.46E-35	8.46E-35
SF037	0.999970129	2.95E-05	2.95E-05
SF038	0.997317782	0.002632368	0.002639447
SF039	0.999994901	2.72E-68	2.72E-68
SF040	0.984213693	0.001748617	0.001776664
SF041	0.967381464	0.032466271	0.033560981
SF042	0.983773998	0.007723998	0.007851395
SF043	0.930536274	0.026087476	0.028034883
SF044	0.986423257	0.013576743	0.013763608

SF045	0.903293009	0.055321489	0.061244234
SF046	1	5.75E-22	5.75E-22
SF047	0.999983907	2.34E-07	2.34E-07
SF048	0.986568777	0.012366753	0.012535115
SF049	0.999996409	3.37E-06	3.37E-06
SF050	0.98664489	0.01335511	0.013535883
SF051	0.992758185	0.007241815	0.007294642
SF052	0.96578616	0.024714668	0.025590207
SF053	0.998167384	0.001832406	0.00183577
SF054	0.999997705	2.80E-21	2.80E-21
SF055	0.982150087	0.017849913	0.018174323
SF056	0.996929205	0.002639034	0.002647163
SF057	0.992615469	0.007384531	0.007439468
SF058	0.990436585	0.009563415	0.009655757
SF059	0.999989049	9.36E-17	9.36E-17
SF060	0.993371503	0.003982738	0.004009313
SF061	1	4.44E-34	4.44E-34
SF062	0.966950986	0.005842767	0.006042465
SF063	0.999942421	3.10E-34	3.10E-34
SF064	0.962550509	0.037370925	0.038824898
SF065	0.937910161	0.062073277	0.06618254
SF066	1	7.48E-15	7.48E-15
SF067	0.999494344	0.000505656	0.000505912
SF068	0.932644525	0.067355475	0.072219879
SF069	0.896569892	0.103430108	0.115362013
SF070	1	1.95E-67	1.95E-67
SF071	1	3.00E-10	3.00E-10
SF072	0.99996505	9.15E-06	9.15E-06
SF073	0.992701258	0.007298742	0.007352405
SF074	0.999266356	3.19E-18	3.19E-18
SF075	0.998249467	0.001750533	0.001753603
SF076	1	1.76E-12	1.76E-12
SF077	0.989593021	0.010406979	0.010516424
SF078	0.999441711	0.000558289	0.000558601
SF079	0.999397735	7.51E-13	7.51E-13
SF080	0.999889619	0.000110381	0.000110394
SF081	0.99131304	0.000455556	0.000459548
SF082	0.999635412	7.30E-07	7.30E-07
SF083	0.999240599	3.47E-10	3.47E-10
SF084	1	4.07E-41	4.07E-41
SF085	0.986122388	0.013877612	0.01407291
SF086	1	1.51E-13	1.51E-13
SF087	1	5.23E-14	5.23E-14
SF088	0.999953286	2.51E-06	2.51E-06
SF089	1	8.33E-14	8.33E-14
SF090	1	1.35E-14	1.35E-14
SF091	1	2.16E-15	2.16E-15
SF092	1	1.36E-31	1.36E-31

SF093	0.999912398	8.76E-05	8.76E-05
SF094	0.974451486	0.007110737	0.007297169
SF095	0.864670674	0.058975597	0.068205848
SF096	0.927655838	0.027259419	0.029385272
SF097	1	2.50E-16	2.50E-16
