# Supplementary material for: Characterization of pathogenic microbiome on removable prostheses with different levels of cleanliness using 2bRAD-M metagenomic sequencing
Source: J Oral Microbiol. 2024 Feb 22;16(1):2317059. doi: 10.1080/20002297.2024.2317059 (PMC10896157; doi:10.1080/20002297.2024.2317059)
Supplement: Appendix 1.pdf [file ZJOM_A_2317059_SM6761.pdf]

Appendix 1

Table 1. Adaptors and primers used for 2bRAD-M library preparation.

| Adaptor | Sequence (5’ to 3’) |
|---|---|
| Adap-1 sense | ACACTCTTTCCCTACACGACGCTCTTCCGATCTNNN |
| Adap-1 antisense | AGATCGGAAGAGC(AminoC6) |
| Adap-2 sense | GTGACTGGAGTTCAGACGTGTGCTCTTCCGATCTNNN |
| Adap-2 antisense | AGATCGGAAGAGC(AminoC6) |
| Primer | |
| Primer1 | ACACTCTTTCCCTACACGACGCT |
| Primer2 | GTGACTGGAGTTCAGACGTGTGCT |
| Primer3 | AATGATACGGCGACCACCGAGATCTACACTCTTTCCC TACACGACGCT |
| Index primer | CAAGCAGAAGACGGCATACGAGATXXXXXXGTGACT GGAGTTCAGACGTGT |
