# Supplementary figures and images for: Characterization of pathogenic microbiome on removable prostheses with different levels of cleanliness using 2bRAD-M metagenomic sequencing
Source: J Oral Microbiol. 2024 Feb 22;16(1):2317059. doi: 10.1080/20002297.2024.2317059 (PMC10896157; doi:10.1080/20002297.2024.2317059)

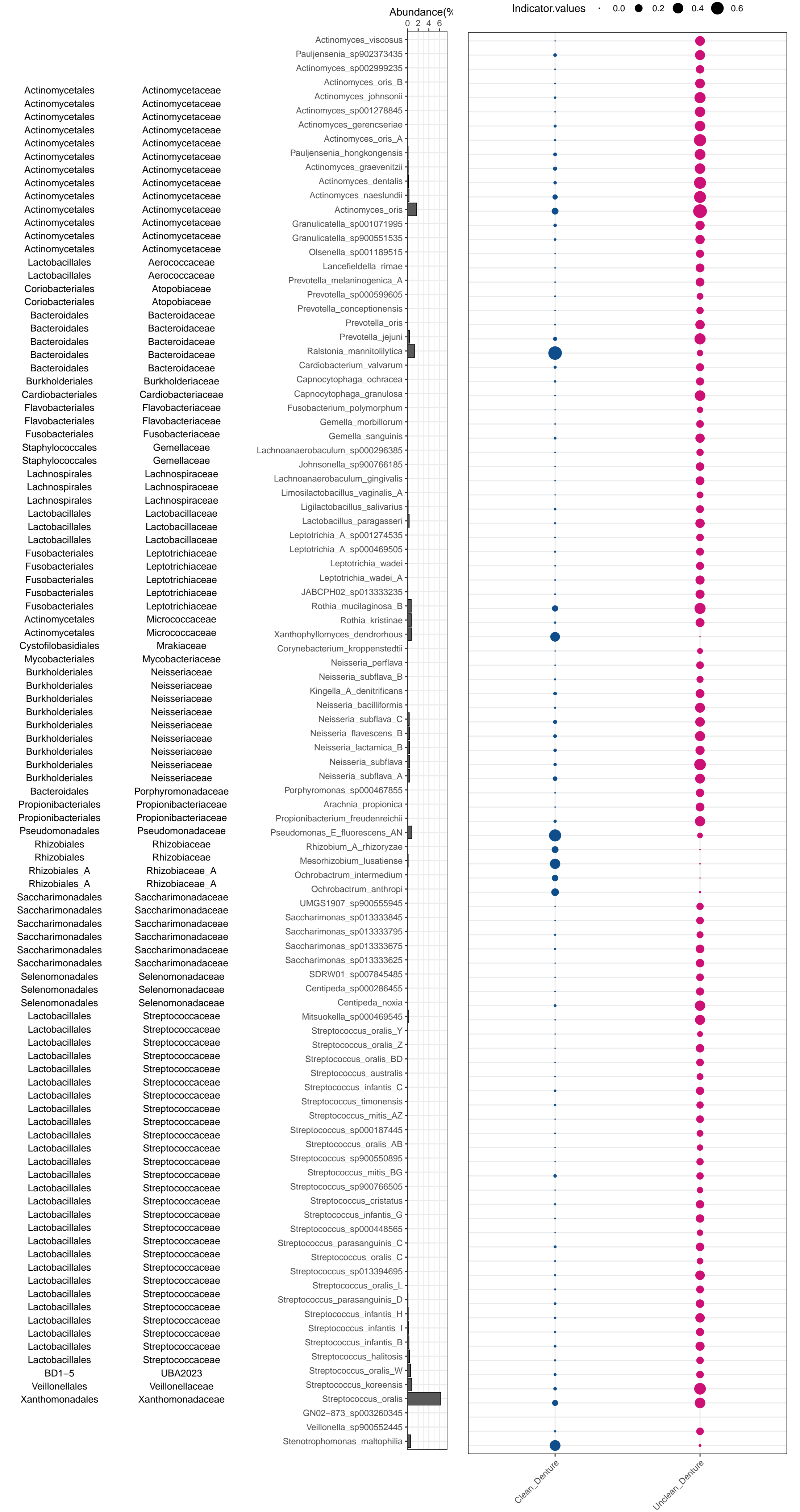

Supplement: Appendix 6.pdf [file ZJOM_A_2317059_SM6760.pdf]

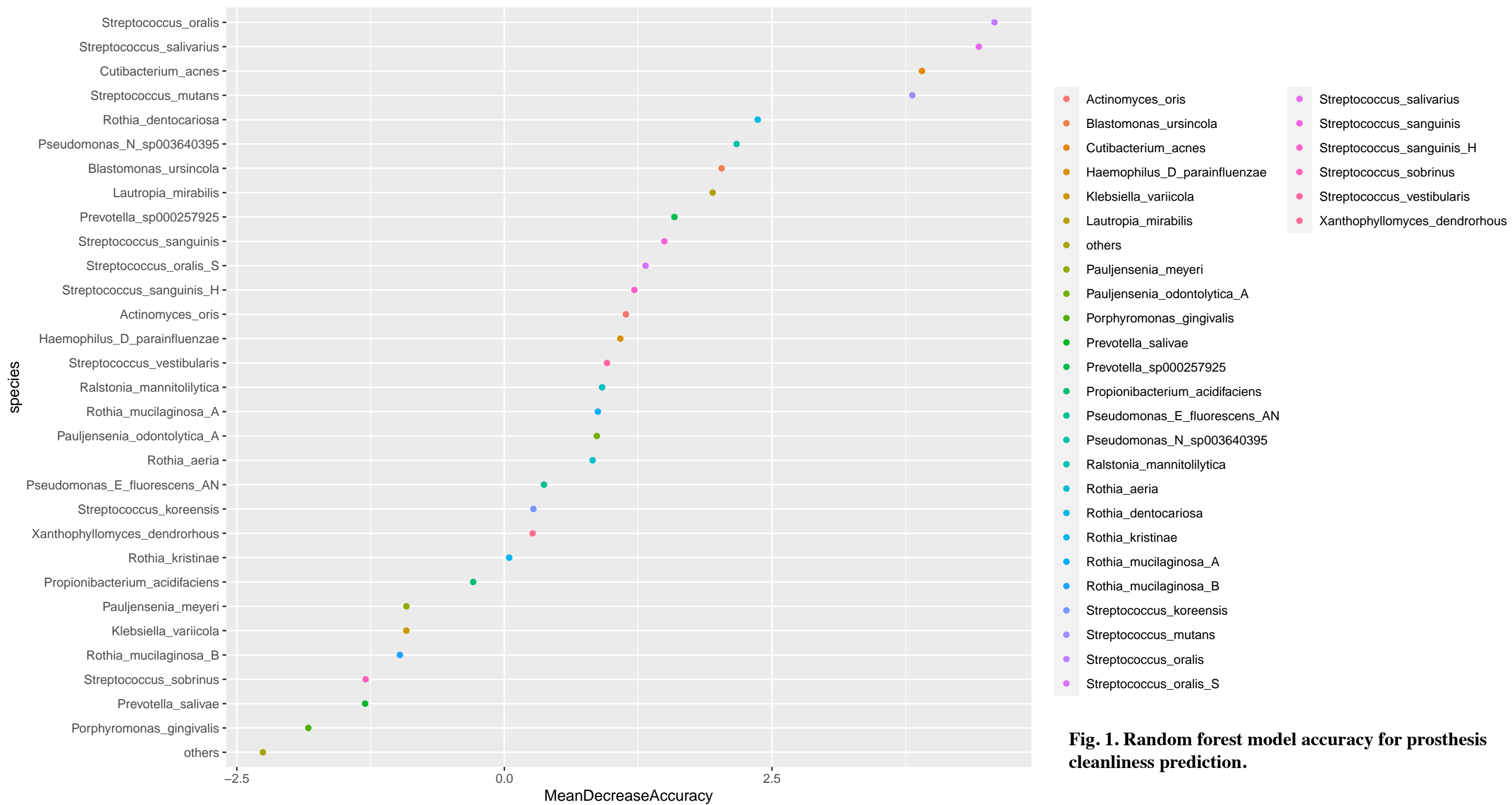

Supplement: Appendix 4.pdf [file ZJOM_A_2317059_SM6759.pdf]

## Appendix 5

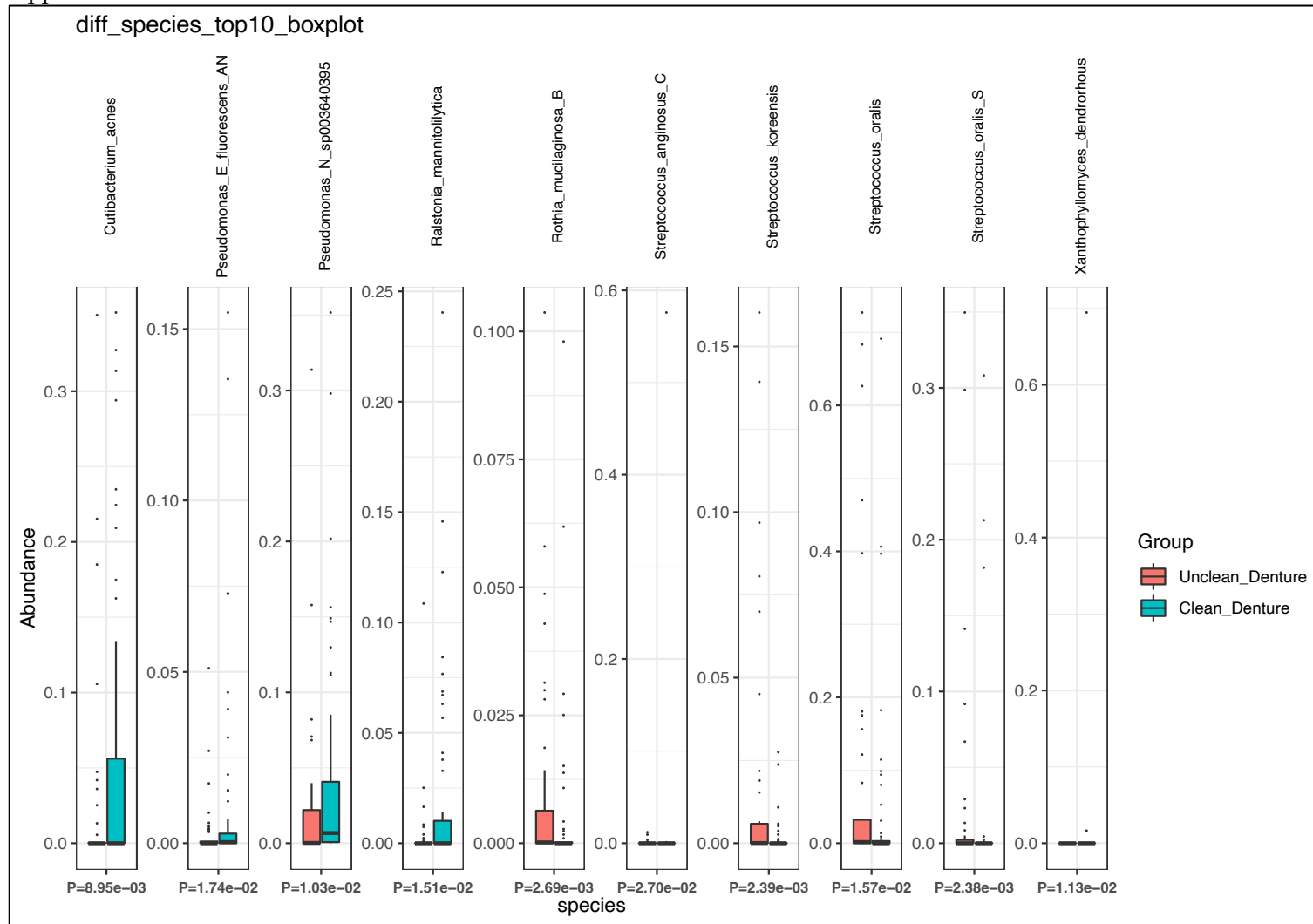

Fig. 1. The top 10 species with different abundances between the two groups.

Supplement: Appendix 5.pdf [file ZJOM_A_2317059_SM6757.pdf]
