# Supplementary material for: Characterization of pathogenic microbiome on removable prostheses with different levels of cleanliness using 2bRAD-M metagenomic sequencing
Source: J Oral Microbiol. 2024 Feb 22;16(1):2317059. doi: 10.1080/20002297.2024.2317059 (PMC10896157; doi:10.1080/20002297.2024.2317059)
Supplement: Appendix 2.pdf [file ZJOM_A_2317059_SM6758.pdf]

Appendix 2:

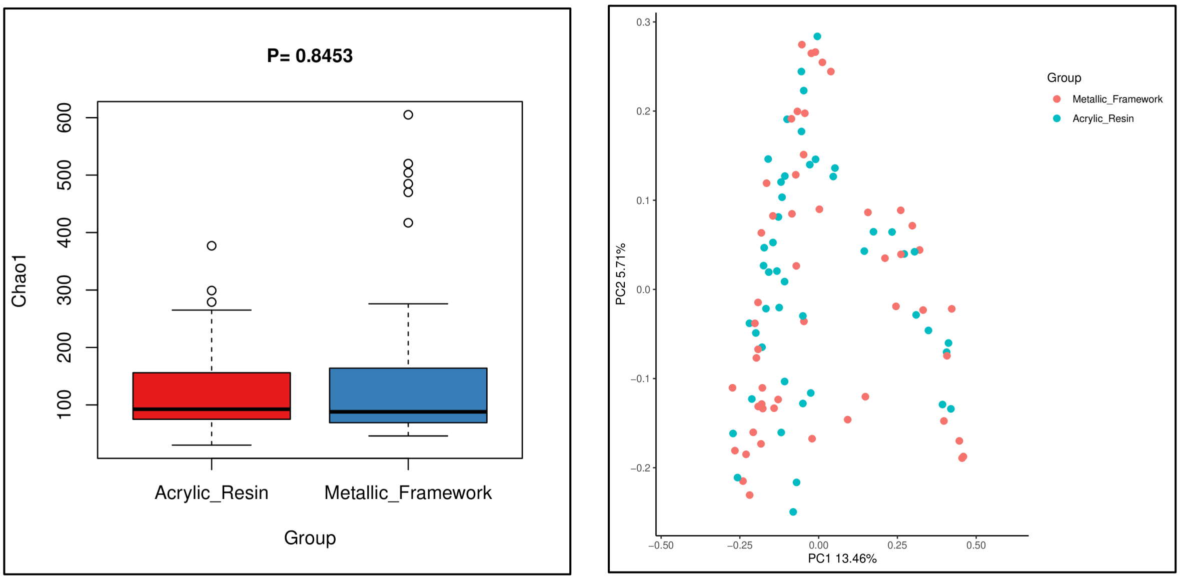

Fig. 1. a) Alpha diversity analysis (Chao 1) revealed no significant difference in microbial richness between clean and unclean prostheses (P = 0.845). b) Beta diversity analysis, Principal Coordinate Analysis based on the Jaccard (PERMANOVA; $R^2 = 0.009$, $P = 0.623$) distance matrix.

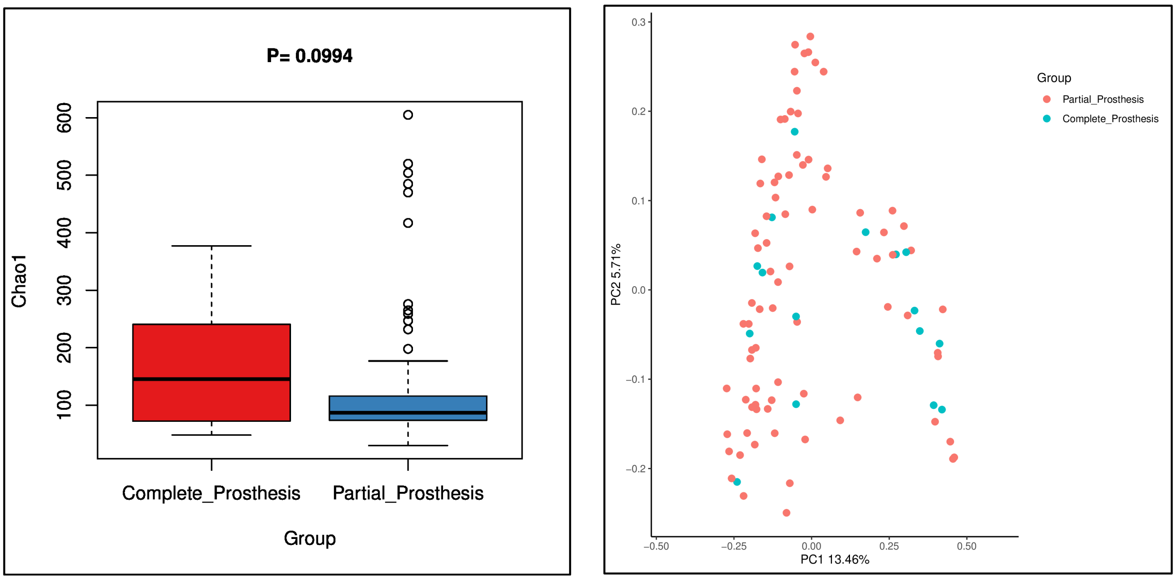

Fig. 2. a) Alpha diversity analysis (Chao 1) revealed no significant difference in microbial richness between complete and partial prostheses (P = 0.099). b) Beta diversity analysis,

Principal Coordinate Analysis based on the Jaccard (PERMANOVA; $R^2 = 0.013$, $P = 0.147$) distance matrix.

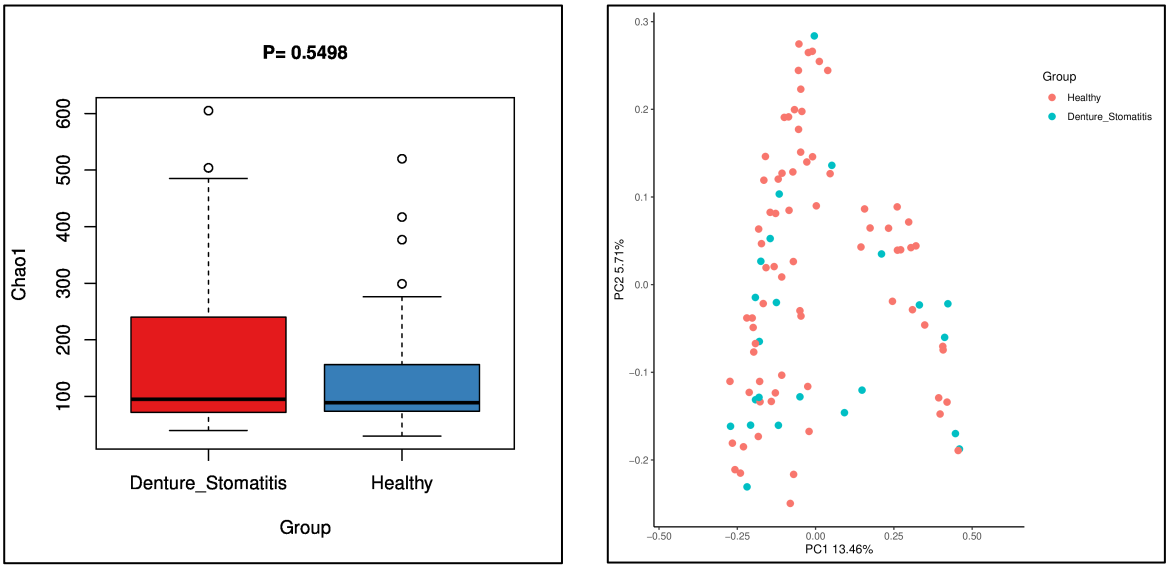

Fig. 3. a) Alpha diversity analysis (Chao 1) revealed no significant difference in microbial richness between denture stomatitis and healthy participants ($P = 0.5498$). b) Beta diversity analysis, Principal Coordinate Analysis based on the Jaccard (PERMANOVA; $R^2 = 0.011$, $P = 0.364$) distance matrix.
